# Assessing decision-making skills with the Script Concordance Test (SCT) in clinical neurology and emergency medicine

**DOI:** 10.1186/s12909-025-06814-7

**Published:** 2025-06-19

**Authors:** Helena-Fee Gudorf, Maximilian Heidrich, Kristoph Rauchstädt, Raphael Scherbaum, Lars Tönges, Anne-Sophie Biesalski

**Affiliations:** 1 Faculty of Medicine, Ruhr University Bochum, Universitätsstrasse 150, Bochum, 44801 Germany; 2 Department of Internal Medicine, Evangelisches Krankenhaus Hattingen, Bredenscheiderstrasse 54, Hattingen, 45525 Germany; 3 Department of Orthopaedics and Trauma Surgery, Knappschaftskrankenhaus Bochum, In Der Schornau 23-25, Bochum, 44892 Germany; 4 Department of Neurology, Ruhr-University Bochum, St. Josef Hospital, Gudrunstraße 56, Bochum, 44791 Germany

**Keywords:** Neurology, Script concordance test, Clinical reasoning, Digital video course, Decision-making Skills, Emergency medicine

## Abstract

**Background:**

Clinical reasoning is an essential medical competence that should be taught and assessed from the beginning of medical studies. These skills can be evaluated using the Script Concordance Test (SCT), which presents daily clinical scenarios characterised by uncertainty. Due to the lack of validated research on this method in Germany, particularly in the field of neurology, we developed and implemented an SCT at Ruhr University Bochum. We compared different teaching methods (clinical seminar vs. digital video course) and their outcomes on the examination.

**Methods:**

A group of 6th-year medical students who had received the same education completed an SCT after participating in either a clinical seminar or a digital video course. The SCT was developed using blueprints on stroke and epilepsy. The test consisted of 40 case vignettes with a total of 120 items. Initially, experts completed the test to establish the reference panel. The final high-stakes examination was created using the aggregate scoring method and an item analysis.

**Results:**

The SCT was completed by 15 experts and 59 students. The final SCT consisted of 112 items and achieved a Cronbach’s alpha of 0.85. A significant difference (*p* < 0.05) was observed between the experts, who achieved a mean score of 81.75, and the students on the first assessment day, who achieved a mean score of 68.92. No significant differences were found between the groups (interactive video course and seminar) or assessment time points. The questionnaire revealed a sense of insecurity in clinical decision-making before the SCT and highlighted the need to incorporate clinical reasoning practices from the beginning of medical studies to mitigate fear in uncertain situations. The SCT helped students structure decision-making processes and and improved their confidence in making decisions.

**Conclusion:**

The SCT is a reliable and valid tool for assessing medical students throughout their university education. Regular exposure to the SCT format would facilitate familiarity with its structure. We propose utilising the SCT as a learning tool rather than solely for assessment purposes. For instance, it could be integrated into teaching methodologies as a think-aloud exercise or incorporated into progress tests.

**Supplementary Information:**

The online version contains supplementary material available at 10.1186/s12909-025-06814-7.

## Background

Clinical reasoning is considered the cornerstone of medical competence and has been described as ‘the sum of the thinking and decision-making processes associated with clinical practice’, although the opinions on this definition vary [1–3]. The key elements of clinical reasoning are data acquisition, problem representation, hypothesis generation, illness script selection, and diagnosis based on knowledge, context, and experience. The internalisation of these elements is crucial to making an accurate diagnosis, preventing diagnostic errors, and thus protecting patient welfare [4–6]. Therefore, clinical reasoning must be taught and assessed from the beginning of medical studies [5, 7]. Because teaching clinical reasoning processes is essential, numerous assessment tools are used to examine different aspects. No method that can evaluate all clinical reasoning steps has yet been developed [3, 8].

Medical teachers must not only teach clinical reasoning skills but also examine their students’ competence in these skills [9, 10].

To achieve this and connect factual knowledge with clinical cases, educators provide students with opportunities to experience patient cases in clinical surroundings [4, 11]. Different formats for teaching clinical reasoning, including the ‘problem-based learning’ or the ‘serial-cue method’, have proven effective [12, 13]. In addition, educators need methods to assess their students’ clinical reasoning skills, one of which is the Script Concordance Test (SCT). The SCT is a valuable tool for evaluating a student's ability to integrate diverse clinical data—such as patient history, presenting symptoms, and diagnostic findings—into a unified and effective decision-making framework which cannot be adequately represented through generalized knowledge alone. Regarding the levels of Miller’s pyramid in clinical reasoning processes, SCTs examine both the ‘knows how’ and the ‘shows how’ levels, improving upon commonly used multiple choice question (MCQ) tests [14]. SCTs examine students’ ability to integrate new clinical information into established illness scripts that represent situations of uncertainty [8, 15]. Illness scripts are organised knowledge structures that are activated in clinical situations and developed throughout a clinician’s entire career [16, 17]. In addition, the test contains a scoring system that accounts for variation in clinicians’ actions in situations of uncertainty due to alternative interpretations [18].

Bernard Charlin et al. first described the SCT in 1999, introducing it as a feasible approach based on script theory to assess data interpretation skills [18]. It can activate, form, and later refine individual illness scripts during medical training, allowing students to encapsulate biomedical knowledge early on and focus on clinically relevant information [19, 20].

The SCT is based on the hypothetico-deductive model [21]. First, a case vignette describes an uncertain clinical scenario. This is followed by new information, each with a specific hypothesis. For each new piece of information and hypothesis, participants must consider how the new information influences the planned hypothesis and whether this hypothesis becomes more or less likely [22]. To assess students’ clinical reasoning skills, the test results are compared with those of experienced clinical experts. Because experts also vary in their SCT responses, all expert choices, even differing ones, are shown in the answer key [18, 23]. This reflects everyday clinical practice, as not all doctors make diagnoses in the same way [24].

The test has been evaluated in different professional fields, such as radiology, urology, neurology, and surgery [25–28]. It represents a validated and reliable method to evaluate students’ clinical reasoning skills compared with the skills of experts [29, 30]. To date, no SCTs in neurology have been published in Germany.

This study developed and implemented an SCT (Table 1) at Ruhr University Bochum to assess whether different clinical reasoning teaching formats directly and measurably affect students’ clinical reasoning abilities. We have based Table 1 on a template by Otterman et al. to provide a clearer overview of our study [31].

**Table 1:** Overview: Script concordance test [31]

Construct to be assessed	Data interpretation in situations of uncertainty
Assessment method	Script Concordance Test
Purpose of the Test	Testing the SCT as a possible assessment method at Ruhr University Bochum
Target group	Medical students in their final year of study
Knowledge domain	Neurology (syncope, epilepsy, and stroke)
Focus of the test	Diagnosis hypotheses, investigation, and treatment options

## Methods

### Development of the SCT

The video course focused on common neurological emergencies, such as epileptic seizures, stroke, and impaired consciousness. Due to the clear diagnostic and therapeutic guidelines for epileptic seizures and stroke, these two areas were selected for the SCT. All participants had successfully completed the second medical exam and an identical medical curriculum, ensuring a comparable level of medical expertise. An expert team consisting of two neurologists, two emergency physicians, and three medical students developed and refined possible hypotheses, investigation strategies, and treatment options for epilepsy and stroke, based on current guidelines as well as content covered in the video course and seminar. Afterwards, two sixth-year medical students revised the SCT and identified potential ambiguities, which were discussed again. The clinical scenarios and items were derived from blueprints established on the basis of material from the German Neurological Society (DGN) guidelines.

### Construction of the SCT

SCT construction followed the guidelines of Fournier et al. and Lubarsky et al. [22, 32]. The obtained material was used to generate 40 common and realistic case vignettes, which were split in half to focus on either epilepsy or stroke. The design was tailored to sixth-year medical students, comprising sections with 12 questions on diagnosis, and 4 each on investigation and therapy [18].

Each case was followed by three items in the first column. New information, such as laboratory values, MRI images, or data from the case history, was added in the second column. As seen in Table 2, the assessment in the last column was conducted using a 5-point Likert scale [33]. The final SCT contained 40 case vignettes, each with three items, as this number is associated with a reliable coefficient [34]. The test was originally developed in German for the assessment of the students and subsequently translated into English.

**Table 2:** a/b/c: Ill-defined patient scenarios described in sample case vignettes, followed by independent items [32, 35]

**Case vignette (Diagnosis question): ** A 24-year-old shopkeeper reported repeated clouding of consciousness. She would suddenly ‘wake up’ and realise that she had just been absent and then had no memory of the duration of the absence.							
Item	If you were thinking of…	And then you find…	This diagnosis becomes…				
B 1	Narcolepsy	… the patient had fallen several times for no reason	−2	−1	0	+1	+2
B 2	Epilepsy	… the patient had two febrile convulsions as a child	−2	−1	0	+1	+2
B 3	Postural orthostatic tachycardia syndrome	… the patient does not suffer from vertigo	−2	−1	0	+1	+2
(−2): Ruled out or almost ruled out. (−1): Less likely. (0): Either more or less likely. (+1): More likely. (+2): Certain or almost certain.							
**Case vignette (Investigation question):** An 18-year-old female patient presented to the neurology outpatient clinic with a suspected epileptic seizure. According to her friends, the episode was characterized by a sudden loss of consciousness accompanied by tonic-clonic movements and enuresis. During the clinical examination, the patient appeared somnolent.							
Item	If you were considering to ask...	And then you find...	This investigation becomes...				
B 4	To conduct an arterial blood gas analysis	the young woman was in a shisha bar with her friends	−2	−1	0	+1	+2
B 5	To order an EEG	the patient's parents have a history of alcohol dependence	−2	−1	0	+1	+2
B 6	To order a CCT	the patient initially fell off a chair	−2	−1	0	+1	+2
(−2): Completely or almost completely unnecessary. (−1): Less useful. (0): Either more or less useful. (+1): More useful. (+2): Completely or almost completely necessary.							
**Case vignette (Treatment question): ** A 24-year-old female patient was admitted to the emergency department following two self-limiting seizure episodes. The next day, she is under your care. Both imaging and EEG findings are unremarkable. You are considering initiating long-term anticonvulsive therapy.							
Item	If you were considering to prescribe...	And then you find...	This prescription becomes...				
B 7	To initiate treatment with Levetiracetam	the patient has a post-traumatic stress disorder	−2	−1	0	+1	+2
B 8	To initiate treatment with Tavor	the patient has an anxiety disorder	−2	−1	0	+1	+2
B 9	To initiate treatment with Lamotrigine	the patient has a poor compliance	−2	−1	0	+1	+2
(−2): Completely or almost completely unnecessary. (−1): Less useful. (0): Either more or less useful. (+1): More useful. (+2): Completely or almost completely necessary.							

### Reference panel

To ensure the assessment's accuracy and its status as a high-stakes examination, 15 to 20 experts are required for the reference panel [23]. In our study, 16 experts took part in the SCT. They were selected based on their expertise in neurological emergencies, though not all were neurologists (some came from emergency medicine). The SCT and its instructions were published on Qualtrics and included questions about the expert’s years of experience, speciality, and professional degree. The experts had three months to complete the computer-based SCT and provide feedback on the assessment tool. Feedback was very positive overall but included minor comments regarding the test's length, clarity, and the use of neurological terms in the case vignettes. Suggestions for improving the SCT instructions were also addressed. We were able to include 15 experts in the reference panel because one expert submitted an incomplete SCT.

### Study design

The only inclusion criterion was being a sixth-year medical student at Ruhr University Bochum. Two different teaching formats were used to assess the effect on students’ clinical reasoning abilities. The medical students were randomised to either an interactive video course or a clinical seminar.

The video course was a digital teaching method that featured case-based video sequences. These sequences were regularly interrupted by the SRA Framework, a decision-making model [36, 37], to actively stimulate the students’ clinical reasoning process. In contrast, the clinical seminars were led by a professor, where students made decisions collaboratively through discussions with each other and in dialogue with the instructor. Both formats covered the same topics, epileptic seizures and stroke, with identical content across the two teaching methods.

Both courses dealt with the topics of epileptic seizures and stroke, as described above. The content was exactly the same, although the type of teaching differed. The video course participants were introduced to the SRA Framework, a decision-making process mode. The virtual case presentations were frequently interrupted by this clinical reasoning process. In the clinical seminars, the students made decisions collaboratively through dialogue. Thus, the instructor only taught in the clinical seminar.

The courses were followed by a written assessment, which consisted of the SCT and a questionnaire. Before starting the SCT, the participants received an oral and written introduction to the method. Three weeks later, they were asked to complete an online follow-up assessment. The SCT was designed to be completed within 90 min, which was considered reasonable [35].

### Questionnaire

The study design included a questionnaire in addition to the SCT. The questionnaire contained questions about participants’ age, gender, medical expertise, and personal opinion of their clinical reasoning level. It included questions rated on a 5-point Likert scale (completely agree to disagree) and free-text questions. The English version of the full questionnaire and the SCT are provided in the supplementary material (DataCollectionForm_SCT.docx).

### Scoring system

The aggregate scoring system was used to account for the various answers chosen by experts, assigning an intrinsic value for each answer. The option on the Likert scale that was selected by the majority was the anchor for the question. The students received 1 point if they chose this answer; if they chose a different answer, a fraction of a point was assigned, which was calculated as the number of experts who chose that answer option divided by the modal value.

The method is illustrated in Table 3 with an example. If 12 of 16 experts chose the response ‘1’ and 4 chose the response ‘2’, then students received one point (12/12) if they chose ‘1’ and 0.33 points (4/12) if they chose ‘2’ [22]. The final SCT score was calculated as a percentage by summing all scores, dividing by the number of items, and multiplying by 100.

**Table 3:** Aggregate scoring system grid

Answers (Example score key for B3 above)	* − *2	* − *1	0	+ 1	+ 2
Number of experts who chose this answer	0	0	0	12	4
Score	0	0	0	12/12	4/12
Points for each possible answer	0	0	0	1	0.33

### Statistical analysis

The test was optimised using R Version 4.2.3 with the psych package for statistical analysis. To maximise internal consistency, all items with negative item discrimination were eliminated This step ensured that the test could effectively distinguish between high- and low-performing students. Internal consistency was measured with Cronbach’s alpha. Descriptive statistics included the mean, maximum and minimum scores, standard deviation, and median. The Shapiro–Wilk test was used to analyse normal distribution, and the Wilcoxon–Mann–Whitney test and Kruskal–Wallis test were used to compare groups. A value of *p* < 0.05 was considered statistically significant.

## Results

A total of 59 sixth-year medical students, the majority of whom were educated in Bochum, participated in the SCT in June 2022. At the time of participation, all students had completed at least one-third of their final year at one of the four university hospitals in Bochum. They also had a comparable level of expertise, having passed the second state examination.

### Construction and item analysis

The final version of the SCT contained 120 items, 112 of which were included after item-total correlation analysis. Initially, we removed six items (15_1, 16_2, 23_2, 25_3, 28_2, and 32_2) that showed negative item discrimination. Following two additional iterations, items 19_1 and 26_2 were removed, resulting in an increase in Cronbach’s alpha from 0.84 to 0.85. All 112 items, which were compared and analyzed as complete items, were then summarised into cases, which were subsequently re-analysed. No further cases had to be eliminated in the subsequent case-based item analysis.

### SCT results

**Fig. 1:**
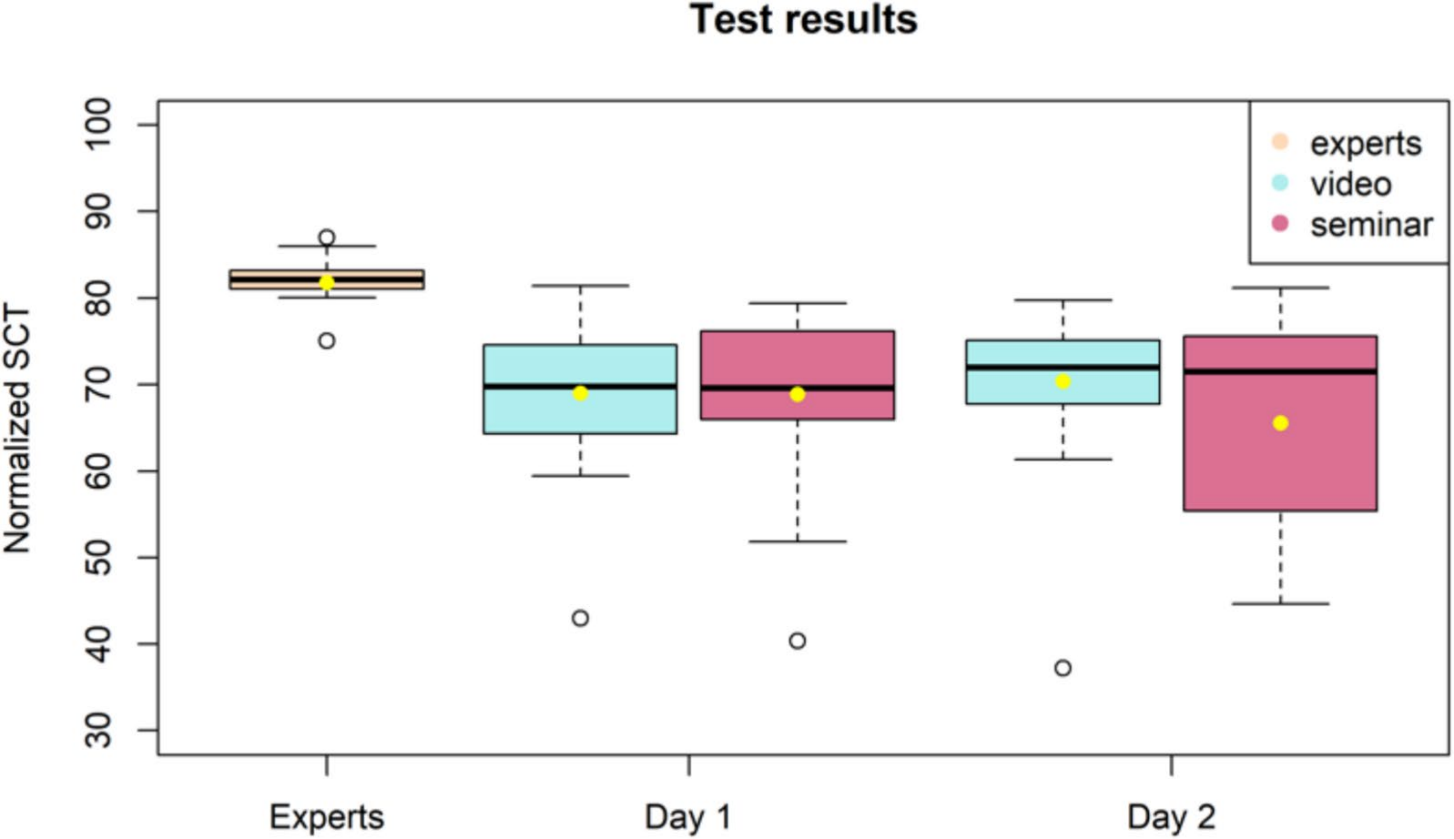
SCT results

Fifteen experts completed the SCT. Table 4 provides the details of the reference panel. On the assessment day, 30 medical students participated in the video course and 29 received a seminar before starting the SCT. We included the participants who completed the follow-up assessment (30 video course participants and 27 seminar participants). SCT results are summarised in Fig. 1 and Table 5. The gender distribution was 42.1% (*n* = 24) males and 57.9% (*n* = 33) females. The participants in the intervention groups had an average age of 27 years. Most participants (94.74%, *n* = 54) were in their last year of studies, and 5.26% (*n* = 3) were in their 5th year. A total of 87.7% (*n* = 50) received their education at Ruhr University Bochum; 12.3% (*n* = 7) finished their studies at another university.

**Table 4:** Reference panel

Question	Answer
Work experience	2–23 (⌀ 8 years)
Professional degree	Senior physicians 5 Specialists 1 Medical assistants 9
Department	Neurology 13 Anaesthesiology 2

**Table 5:** SCT results

*Group*	***Mean***	*Min*	*Max*	*Median*	*Σ*	*Cronbach’s alpha*
*Experts*	**81.75**	75.04	86.97	82.14	3.31	0.85
*Video Day 1*	**68.98**	42.96	81.43	69.75	7.68	
*Seminar Day 1*	**68.86**	40.36	79.38	69.58	9.06	
*Video Follow-up*	**70.32**	37.25	79.72	71.97	7.94	
*Seminar Follow-up*	**65.52**	44.66	81.18	71.45	12.21	

The Shapiro–Wilk test showed no significant differences between groups (*p* > 0.05), and the Wilcoxon–Mann–Whitney test revealed significant differences between students and experts (*p* < 0.05). No significant differences were observed between the groups (interactive video course and seminar) or time points when the participants completed the SCT. The Kruskal–Wallis test showed no significant differences between items related to diagnosis, investigation, or therapy. The Wilcoxon–Mann–Whitney test showed no significant difference between the topics of epilepsy and stroke and the level of expertise (*p* = 0.258).

### Questionnaire results

Fifty-four participants answered the free-text question ‘How have you made clinical decisions so far?’ (27 from the video course and 27 from the seminar). Most of the participants stated that they made decisions by experience (*n* = 12) or by knowledge (*n* = 8). Some said that their intuition guided them (*n* = 5). Content analysis revealed that two participants in the video course group and eight in the seminar group said that they never had to make decisions independently. Four participants in the seminar group provided positive feedback about the course, such as ‘I was often conservative, but now I am more confident’ and ‘The course gave me certainty regarding stroke diagnosis’. The other question assessed changes in decision-making after the course. Thirty-three participants answered this question, and twelve reported positive changes. For example, ‘…the course outlined the structure again and simplified the process’ and ‘…the course provides confidence to trust my own decisions’.

### Results content analysis

**Fig. 2:**
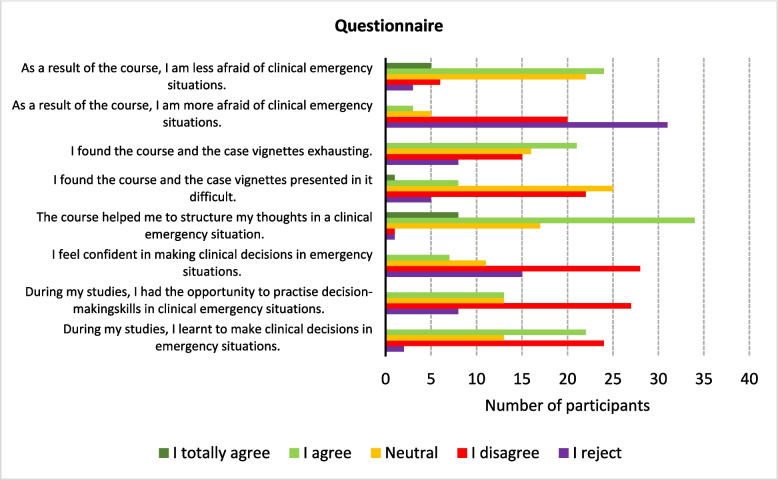
Questionnaire results

Most (68.9%, *n* = 42) of the students agreed or completely agreed that the course helped them with structuring their thoughts in clinical emergencies; 66.7% of them completed the interactive video course. In addition, 70.5% disagreed with or rejected the statement ‘I feel secure when making decisions in neurological emergencies’. Finally, 48.3% (*n* = 29) agreed or completely agreed with the statement ‘I have less fear after the course’, of whom 65.5% (*n* = 19) were assigned to the video course. The results of the Likert scale portion of the questionnaire are presented in Fig. 2.

## Discussion

This study developed and tested the SCT assessment method at Ruhr University Bochum and used it to compare two methods of teaching clinical reasoning.

We found only small differences in students’ assessed clinical reasoning skills between the two different teaching methods (interactive video course vs. clinical seminar). However, we gained important insights into the possible uses and pitfalls of the SCT.

### Validity

SCTs are frequently tested and used on an international level. They show reliable and valid results regarding respondents’ competence in interpreting clinical data in situations of uncertainty [30]. Lubarsky analysed five categories, which we used as a guide. The first category focuses on content. Therefore, we excluded all items for which the answers were not clustered around the most common expert answer and focused on important teaching knowledge. The second category, response process, emphasises that a higher expertise level correlates with a higher SCT score. Experts had a significantly higher SCT score than students, which shows the correct application of the SCT.

Regarding the third category, internal structure, most established SCTs had Cronbach’s alpha values of 0.70–0.90 and used 15–20 experts for reliability. Our study showed a Cronbach’s alpha of 0.85 and involved 15 experts. The fourth category, relations to other variables, focuses on comparing MCQ tests and SCTs. Some studies have aimed to compare SCTs with MCQ tests, but no standardised recommendation has been established due to the difficulty of comparability. We focused on feedback from the students. The SCT resolved the ‘one-best-answer’ problem, which arises in situations of uncertainty with no single correct answer, as clinicians weigh aspects differently and do not handle situations in the same way [18, 38].

The last category, consequences, highlights that the aggregate method is the ‘key determinant of discriminatory power’. For this reason, we applied this method.

### Group recruitment

Because this assessment method is still uncommon, most experts were not familiar with it; therefore, several questions arose, which could be addressed by the instructions. As shown in Table 4, we aimed to include a panel diverse in work experience and academic degrees while ensuring uniform expertise in neurological emergencies. Therefore, in addition to neurologists, anaesthesiologists were also included in the panel to ensure comprehensive coverage of the field of neurological emergencies.

There are no established recommendations regarding the ideal size of the participant group. We were able to recruit a homogeneous group of 59 sixth-year medical students, all of whom had completed the same medical education and having passed the second state examination. The small sample size may have contributed to the lack of significant results. The content of the clinical vignettes and items is valid and suitable for sixth-year medical students, as they have just passed the second state examination and should have the necessary knowledge.

### Comparison of results

This study developed an SCT to assess and compare students who participated in an interactive video course and those who took part in a clinical seminar. Although students did not differ significantly on the initial assessment day (68.98 (Σ = 7.68) vs. 68.86 (Σ = 9.06), the follow-up results showed that the video course participants achieved a slightly higher mean score (70.32 (Σ = 9.94) vs. 65.52 (Σ = 12.21)). Additionally, the Shapiro–Wilk test revealed no significant differences between the groups (*p* > 0.05), while the Wilcoxon-Mann–Whitney test showed minimal significant differences between students and experts (*p* < 0.05). Engaging with the decision-making process model may have helped these participants structure their thought processes and apply them in the SCT, leading to improved performance. We also considered the potential influence of prior work experience, particularly nursing, on clinical decision-making. However, because nearly all participants had nursing experience, this likely did not significantly impact the results. This suggests that the SCT can be used as an assessment method, as working experience as a nurse does not confer a relevant advantage. Additionally, the SCT can be administered as an online exam, as the answers are not easily found on the internet. Notably, assessing clinical reasoning through a single SCT likely has a limited impact on enhancing this skill. One similar study compared the SCT results of students who were presented with online virtual patient cases or classroom lectures. The lack of significant differences in the students’ results indicates that virtual case presentations can be used during pandemics, cancelled lectures, or shortened internships [39]. This should not become common practice, but it allows students to acquire knowledge to establish it through experience in the clinic.

### Student feedback

Regarding the content analysis, more students in the seminar group reported never needing to make decisions independently. This might indicate that the interactive nature of the video course allowed the participants to feel more included in the decision-making process. It is unfortunate that most sixth-year medical students still feel insecure about making decisions in neurological emergencies. Almost 50% agreed or completely agreed with the statement ‘I have less fear after the course’, which indicates that students have low confidence in emergency situations and reveals the importance of teaching clinical reasoning. Teachers must encourage students to think clinically and provide them with tools such as decision-making process models and case vignettes. Of the 29 students who reported feeling less concerned after the course, 20 participated in the video course. These students may have felt directly addressed in the video course, unable to conceal their insecurity as they might have in the seminar. The allocated processing time of 90 min for 120 items was more than sufficient, as no student needed the full duration.

### Limitations

One key limitation was that the students were not familiar with the SCT format. Leclerc et al. mentioned that it is challenging to adapt to an alternative assessment method. Nevertheless, experts believe that this exam format reflects everyday situations accurately and is superior to the MCQ test [40, 41]. Establishing an SCT involves more effort than setting up an MCQ test; for an SCT blueprints must be formulated, along with case vignettes and items. Another challenge in constructing an SCT is finding an expert group suitable for the various topics with the time to participate. Furthermore, the cohort of 59 students can lead to several limitations. It may reduce the statistical power of, diminishing its ability to detect significant effects, and it may not be fully representative of the entire target population. Due to efforts to avoid bias, we unfortunately did not have the opportunity to include more students in the study. Additionally, clinical teachers must invest time in discussing thought processes and helping students structure their illness scripts. However, once the SCT has been created and validated, it can be used repeatedly. This involves working on a topic once and then providing students with a learning method that can be used offline in seminars and online. The SCT also avoids the cueing effect problem of MCQ tests [42]. Overall, clinical reasoning cannot be taught and learned solely online; in-person interaction is essential.

The practice of pass-or-fail grading in SCT assessments has not yet gained widespread use. Duggan et al. described their grading system as follows: an A grade was given up to − 2 standard deviations from the mean of the experts, a B grade up to − 3 standard deviations, a C grade up to − 4 standard deviations, and an initial fail below − 4 standard deviations [43]. Because the expertise of the panel of experts influences the evaluation, we believe that using a pass/fail threshold is not suitable. Applying such a threshold in our study would have resulted in only 33 out of 59 students (56%) passing, as the standard deviation of the experts was 3.31.

### Future research

Many students in their final year of medical school experience insecurity when making independent decisions. Helping them feel more confident in uncertain situations and teaching them how to organise their thoughts is important as they begin working as medical assistants. Enhancing learning through discussions of clinical cases in seminars may significantly improve their skills. Therefore, we would recommend using the SCT as a basis for group discussions in medical education. Additionally, residents, who often seek to mentor medical students, can combine the ‘think aloud method’ with the SCT to gain insight into their thought processes [44, 45]. This represents an opportunity for medical students as well as residents. No published articles show evidence of residents with less working experience or the usage of the ‘think aloud method’ in bedside teaching.

Various methods of script activation could be used to gain insights into individual decision-making processes. This approach can assist students in bridging their factual knowledge with clinical encounters. Given the critical importance of preventing diagnostic errors in situations of uncertainty, we contend that the implementation of the SCT aligns well with emergency medicine; in which speed is crucial, and situations are often stressful and complex. In this field, topics can be effectively divided into diagnosis, investigation, and therapy questions.

The response to the video course was generally more positive than to the seminar, possibly because students, as ‘digital natives’, were more enthusiastic and familiar with digital learning methods.

A hybrid format is feasible for video courses, allowing educators to prepare videos and assemble content themselves. Additionally, the SCT could serve as a digital learning tool to compensate for the lack of bedside teaching during situations such as the COVID-19 pandemic [46].

## Conclusion

Because the SCT is feasible to use in workshops and can improve clinical reasoning skills, we recommend the incorporation of the developed SCT in seminars or bedside teaching at Ruhr University. Including the SCT at the beginning of the curriculum could help students encapsulate knowledge early, identify areas that must be revised, and develop illness scripts to equip students with essential skills. In addition, exchanging thought processes promotes self-reflection and avoids cognitive errors. MCQs only test factual knowledge and, therefore, do not adequately prepare medical students for situations of uncertainty. Our recommendations are also applicable to other universities that aim to support hands-on training in medical education although the approach was only implemented at Ruhr University. Although the SCT is often viewed critically in the literature, we have had very positive experiences with it. Once the test is developed, it can be used frequently and prepares students much better for practical everyday situations.

In conclusion, we recommend the integration of the developed SCT into seminars or bedside teaching during medical studies to enhance clinical reasoning skills. Early inclusion of the SCT coupled with its longitudinal application in the curriculum can aid in early knowledge retention, the identification of areas for revision, and the development of illness scripts to equip students with essential skills. Unlike MCQ tests, which primarily assess factual knowledge, the SCT is better suited for preparing medical students for uncertain situations. Considering the SCT for inclusion in progress tests would offer the opportunity to assess both the ‘know’ and ‘knows how’ levels of Miller’s pyramid from an early stage, allowing for annual feedback on decision-making skills [47]. Other options include combining SCT with the written ‘think aloud’ method, facilitating comparison between student and expert thought processes, and combining the SCT with the CTA method, allowing students to work through their thoughts while answering items, thus promoting more structure in uncertain situations [45, 48].

## Supplementary Information

Supplementary Material 1.

## Data Availability

The full data can be requested at any time and released to interested parties. Therefore please send an Email to anne-sophie.biesalski@klinikum-bochum.de. Unfortunately, the data are not available in an online repository for data protection reasons.

## Abbreviations

SCT: Script Concordance Test
RUB: Ruhr University Bochum
