# Supplementary material for: Assessing decision-making skills with the Script Concordance Test (SCT) in clinical neurology and emergency medicine
Source: BMC Med Educ. 2025 Jun 19;25:852. doi: 10.1186/s12909-025-06814-7 (PMC12180276; doi:10.1186/s12909-025-06814-7)
Supplement: Supplementary file 1 — Supplementary Material 1. [file 12909_2025_6814_MOESM1_ESM.docx]

Data collection form

**VERSION 1, 28.06.2022**

Dear participant,

thank you for your participation in this study! In order to be able to understand in which study arm you participated and how sustainable the respective teaching format has affected your knowledge and skills, we ask you to complete this data collection form today and to participate in the online survey in moodle in three weeks' time. It is necessary to complete both surveys in order to receive the expense allowance.

As the data is stored anonymously (without the possibility of assigning it to actual users or accounts), a so-called ‘personal code’ is required in order to be able to assign the data of the survey dates to each other and at the same time maintain the anonymity of all participants.

The personal code consists of a combination of letters and numbers that is not known to anyone at the university apart from you, but which you can always derive yourself.

**The personal code is made up of the following five letters or numbers:**

- The first two letters of the name of your primary school (for names such as St Mauritius School, please use MA)
- First letter of the mother's first name
- Month of birth as a numerical number (e.g. May = 05)

Please enter the resulting code (e.g.: name of primary school ‘Hubertusschule’, mother's name ‘Bettina’, month of birth ‘December’ = ‘HUB12’) in the grey boxes on each study sheet.

Personal Code:

Which teaching format was used?

Interactive video course (digital) Clinical case seminar

**Examination Part B**

Personal Code:

Script-Concordance-Test

The Script Concordance test assesses students' ability to make decisions in clinical settings. Their results are compared with those of professionals (experts) and thus provide and insight into the students' decision-making skills.

Explanation of how to take the Script Concordance Test
The Script Concordance Test consists of different clinical case vignettes. The case vignette contains the brief, most important clinical information. Each case vignette is followed by three items, each consisting of three parts.

In the first column, "If you were thinking of," you will find hypotheses that represent the suspected diagnoses. In the second column, "And then learn/find that..." you will be given a new piece of information about the case, which you are to evaluate in the third part. What does this new information do to the hypothesis (i.e. its suspected diagnosis or the planned diagnosis or therapy)?

Example: You suspect that the patient has a myocardial infarction (hypothesis) and then find out that the patient also has untreated arterial hypertension (new information). Does this make their suspected diagnosis ("myocardial infarction") more likely or less likely? Please mark the respective possibility on the Likert scale accordingly, e.g., less probable (-1), neither less or more probable (0), or more probable (+1). Is it even possible that the hypothesis is ruled out (-2) or certained (+2) by the new information?

The classification of a therapy or diagnosis as "probable", "certained", etc. always refers to the additional information, not to the information from the case vignette. Example: The case vignette reveals that a patient is suspected of having a myocardial infarction. Consideration is given to writing an ECG. As additional information, it is revealed that the patient's grandmother's name is Marianne. This information has no bearing on whether or not an ECG is appropriate. Therefore, the middle option " neither less or more probable " would be selected (although, of course, an ECG is the correct measure in the case of a suspected myocardial infarction).

Please note that the different items (=lines) in the case vignettes do NOT refer to each other and do not build on each other. Each case vignette therefore contains three different suspected diagnoses, each of which must be reviewed and answered with its own new information.

The case vignettes can review diagnoses, investigations, and treatment decisions. The Likert scale adapts to each of these topics.

Please check the box that makes the most sense to you – on the background of your own clinical experience. This is not an examination and the questions are deliberately chosen so that even an experienced physician probably does not have all the background information “down pat" at the moment.

**Example (Diagnosis type)**

| **Case vignette A: A 23-year-old female patient is brought to the emergency department by the ambulance service with unclear unconsciousness.** | | | | | | | | |
| --- | --- | --- | --- | --- | --- | --- | --- | --- |
| Item number | If you were thinking of… | And then you find… | This hypothesis becomes… | | | | | |
| B | Alcohol intoxication | a tongue bite is present | -2 | -1 | 0 | +1 | +2 |  |
| B | Convulsive syncope | the convulsion lasted 10min | -2 | -1 | 0 | +1 | +2 |  |
| B | Psychogenic seizure | the eyes were closed | -2 | -1 | 0 | +1 | +2 |  |

**Explanation of the Likert scale (diagnosis type):**

| -2 | Ruled out or almost ruled out |
| --- | --- |
| -1 | Less probable |
| 0 | Neither less or more probable |
| +1 | More probable |
| +2 | Certain or almost certain |

To explain the first item:

My suspected diagnosis is alcohol intoxication. If I now find out that the patient has a tongue bite, my diagnosis is then almost ruled out (-2), less probable (-1), neither less or more probable (0), more probable (+1), or almost certain (+2). For the other two items, the hypothesis must also be entered.

Case 1

| **Case vignette 1:**  **A 45-year-old patient is brought with unclear unconsciousness to the emergency department by paramedic. The patient slowly gets back his consciousness and is oriented times 4.** | | | | | | | | | | | |
| --- | --- | --- | --- | --- | --- | --- | --- | --- | --- | --- | --- |
| Item number | | | If you were thinking of… | And then you find... | | This hypothesis becomes... | | | | | |
| B 1 | | | Generalized tonic-clonic seizure | the patients lateral tongue bite | | -2 | -1 | 0 | +1 | +2 |  |
| B 2 | | | Convulsive syncope | the patient has complained of nausea shortly before the event | | -2 | -1 | 0 | +1 | +2 |  |
| B 3 | | | Psychogenic seizure | there were initial rhythmic discharges of the extremities for several minutes | | -2 | -1 | 0 | +1 | +2 |  |
| -2 | Ruled out or almost ruled out | | |  |  |  |  |  |  |  |  |
| -1 | Less probable | | |  |  |  |  |  |  |  |  |
| 0 | Neither less or more probable | | |  |  |  |  |  |  |  |  |
| +1 | More probable | | |  |  |  |  |  |  |  |  |
| +2 | Certain or almost certain | | |  |  |  |  |  |  |  |  |

| **Case vignette 2:**  **A 19-year-old trainee became unconscious in the patient room and fell. She regained consciousness after about a minute. She has no memory of the fall and cannot say how long she was laying on the floor.** | | | | | | | | | | | |
| --- | --- | --- | --- | --- | --- | --- | --- | --- | --- | --- | --- |
| Item number | | | If you were thinking of… | And then you find... | | This hypothesis becomes... | | | | | |
| B 4 | | | Generalized tonic-clonic seizure | the trainee has no injuries | | -2 | -1 | 0 | +1 | +2 |  |
| B 5 | | | Convulsive syncope | the trainee had myocloniform discharges shortly after the fall | | -2 | -1 | 0 | +1 | +2 |  |
| B 6 | | | Psychogenic seizure | Tthe eyes were open during the seizure | | -2 | -1 | 0 | +1 | +2 |  |
| -2 | Ruled out or almost ruled out | | |  |  |  |  |  |  |  |  |
| -1 | Less probable | | |  |  |  |  |  |  |  |  |
| 0 | Neither less or more probable | | |  |  |  |  |  |  |  |  |
| +1 | More probable | | |  |  |  |  |  |  |  |  |
| +2 | Certain or almost certain | | |  |  |  |  |  |  |  |  |

| **Case vignette 3:**  **A 35-year-old female patient arrives at the emergency department. Her partner reports that the patient passed out in the apartment. This had happened several times before. However, there was never any clinical clarification.** | | | | | | | | | | | |
| --- | --- | --- | --- | --- | --- | --- | --- | --- | --- | --- | --- |
| Item number | | | If you were thinking of… | And then you find... | | This hypothesis becomes... | | | | | |
| B 7 | | | Atrioventricular Block | that the unconsciousness was self-limiting | | -2 | -1 | 0 | +1 | +2 |  |
| B 8 | | | Epileptic seizure | that the patient has type 1 diabetes mellitus and has had multiple hypoglycemic episodes in the past | | -2 | -1 | 0 | +1 | +2 |  |
| B 9 | | | Micturition syncope | the patient wanted to go to the toilet before the event | | -2 | -1 | 0 | +1 | +2 |  |
| -2 | Ruled out or almost ruled out | | |  |  |  |  |  |  |  |  |
| -1 | Less probable | | |  |  |  |  |  |  |  |  |
| 0 | Neither less or more probable | | |  |  |  |  |  |  |  |  |
| +1 | More probable | | |  |  |  |  |  |  |  |  |
| +2 | Certain or almost certain | | |  |  |  |  |  |  |  |  |

| **Case vignette 4:**  **A 45-year-old patient is brought to the emergency room. From the handover, you have received the information that the patient was found on the sidewalk and laid on the ground without moving for several minutes.** | | | | | | | | | | | |
| --- | --- | --- | --- | --- | --- | --- | --- | --- | --- | --- | --- |
| Item number | | | If you were thinking of… | And then you find... | | This hypothesis becomes... | | | | | |
| B 10 | | | Hypoglycemia | the patient has bilateral dilated pupils that are barely sensitive to light | | -2 | -1 | 0 | +1 | +2 |  |
| B 11 | | | Orthostatic syncope | a systolic blood pressure of 105 mmHg | | -2 | -1 | 0 | +1 | +2 |  |
| B 12 | | | Intoxication | the patient has isochoric, moderately wide pupils with direct and indirect light responsiveness | | -2 | -1 | 0 | +1 | +2 |  |
| -2 | Ruled out or almost ruled out | | |  |  |  |  |  |  |  |  |
| -1 | Less probable | | |  |  |  |  |  |  |  |  |
| 0 | Neither less or more probable | | |  |  |  |  |  |  |  |  |
| +1 | More probable | | |  |  |  |  |  |  |  |  |
| +2 | Certain or almost certain | | |  |  |  |  |  |  |  |  |

| **Case vignette 5:**  **A 70-year-old pensioner is brought to the hospital by the ambulance service from a retirement home. She was lying on the floor when she was found by the nursing staff. The lying time is not known.** | | | | | | | | | | | |
| --- | --- | --- | --- | --- | --- | --- | --- | --- | --- | --- | --- |
| Item number | | | If you were thinking of… | And then you find... | | This hypothesis becomes... | | | | | |
| B 13 | | | Syncope | an enuresis has occurred | | -2 | -1 | 0 | +1 | +2 |  |
| B 14 | | | Craniocerebral trauma | the left pupil is wider than the right pupil | | -2 | -1 | 0 | +1 | +2 |  |
| B 15 | | | Cryptogenic fall attack (also: "drop attack") | the event occurred without a loss of consciousness | | -2 | -1 | 0 | +1 | +2 |  |
| -2 | Ruled out or almost ruled out | | |  |  |  |  |  |  |  |  |
| -1 | Less probable | | |  |  |  |  |  |  |  |  |
| 0 | Neither less or more probable | | |  |  |  |  |  |  |  |  |
| +1 | More probable | | |  |  |  |  |  |  |  |  |
| +2 | Certain or almost certain | | |  |  |  |  |  |  |  |  |

| **Case vignette 6:**  **The mother of a 28-year-old pregnant woman called the emergency services. She said that the pregnant woman fell in the doorway of her house and fell to the ground. She was pale and regained consciousness after a few seconds, she said.** | | | | | | | | | | | |
| --- | --- | --- | --- | --- | --- | --- | --- | --- | --- | --- | --- |
| Item number | | | If you were thinking of… | And then you find... | | This hypothesis becomes... | | | | | |
| B 16 | | | Cardiac syncope | the event occurred immediately after getting out of bed | | -2 | -1 | 0 | +1 | +2 |  |
| B 17 | | | Vasovagal syncope | the patient had a fixed gaze and nibbled before the event | | -2 | -1 | 0 | +1 | +2 |  |
| B 18 | | | Orthostatic syncope | the patient drank a glass of cold water before the event | | -2 | -1 | 0 | +1 | +2 |  |
| -2 | Ruled out or almost ruled out | | |  |  |  |  |  |  |  |  |
| -1 | Less probable | | |  |  |  |  |  |  |  |  |
| 0 | Neither less or more probable | | |  |  |  |  |  |  |  |  |
| +1 | More probable | | |  |  |  |  |  |  |  |  |
| +2 | Certain or almost certain | | |  |  |  |  |  |  |  |  |

| **Case vignette 7:**  **A 48-year-old patient arrives at the emergency department by ambulance. In the handover, dyspnea and chest pain are reported.** | | | | | | | | | | | |
| --- | --- | --- | --- | --- | --- | --- | --- | --- | --- | --- | --- |
| Item number | | | If you were thinking of… | And then you find... | | This hypothesis becomes... | | | | | |
| B 19 | | | Pulmonary artery embolism | the patient was initially unconscious for a short time | | -2 | -1 | 0 | +1 | +2 |  |
| B 20 | | | Pulmonary artery embolism | the Wells score is 5 points | | -2 | -1 | 0 | +1 | +2 |  |
| B 21 | | | Pulmonary artery embolism | the patient has a temperature of 37.8°C | | -2 | -1 | 0 | +1 | +2 |  |
| -2 | Ruled out or almost ruled out | | |  |  |  |  |  |  |  |  |
| -1 | Less probable | | |  |  |  |  |  |  |  |  |
| 0 | Neither less or more probable | | |  |  |  |  |  |  |  |  |
| +1 | More probable | | |  |  |  |  |  |  |  |  |
| +2 | Certain or almost certain | | |  |  |  |  |  |  |  |  |

| **Case vignette 8:**  **A 60-year-old secretary has just been taken to the emergency room. She had suddenly fallen from her office chair with her eyes closed. Now, she is very worried and complains of dyspnea.** | | | | | | | | | | | |
| --- | --- | --- | --- | --- | --- | --- | --- | --- | --- | --- | --- |
| Item number | | | If you were thinking of… | And then you find... | | This hypothesis becomes... | | | | | |
| B 22 | | | Pulmonary artery embolism | the patient returned from a long vacation trip three days ago | | -2 | -1 | 0 | +1 | +2 |  |
| B 23 | | | Epileptic seizure | the patient consumes alcohol regularly | | -2 | -1 | 0 | +1 | +2 |  |
| B 24 | | | Aortic valve stenosis | the patient has a history of arterial hypertension | | -2 | -1 | 0 | +1 | +2 |  |
| -2 | Ruled out or almost ruled out | | |  |  |  |  |  |  |  |  |
| -1 | Less probable | | |  |  |  |  |  |  |  |  |
| 0 | Neither less or more probable | | |  |  |  |  |  |  |  |  |
| +1 | More probable | | |  |  |  |  |  |  |  |  |
| +2 | Certain or almost certain | | |  |  |  |  |  |  |  |  |

| **Case vignette 9:**  **A 33-year-old patient went for a run in the park and suddenly fell to the ground. Eyewitnesses report that he "jerked" a few times and then "lay there as if he was lifeless".** | | | | | | | | | | | |
| --- | --- | --- | --- | --- | --- | --- | --- | --- | --- | --- | --- |
| Item number | | | If you were thinking of… | And then you find... | | This hypothesis becomes... | | | | | |
| B 25 | | | Status epilepticus | the patient's pupils are anisochoric | | -2 | -1 | 0 | +1 | +2 |  |
| B 26 | | | Status epilepticus | the jaw was firmly closed during the event | | -2 | -1 | 0 | +1 | +2 |  |
| B 27 | | | Status epilepticus | the patient is now somnolent | | -2 | -1 | 0 | +1 | +2 |  |
| -2 | Ruled out or almost ruled out | | |  |  |  |  |  |  |  |  |
| -1 | Less probable | | |  |  |  |  |  |  |  |  |
| 0 | Neither less or more probable | | |  |  |  |  |  |  |  |  |
| +1 | More probable | | |  |  |  |  |  |  |  |  |
| +2 | Certain or almost certain | | |  |  |  |  |  |  |  |  |

| **Case vignette 10:**  **A 24-year-old shopkeeper reported repeated clouding of consciousness. She would suddenly "wake up" and realize that she had just been absent and then had no memory of the duration of the absence.** | | | | | | | | | | | |
| --- | --- | --- | --- | --- | --- | --- | --- | --- | --- | --- | --- |
| Item number | | | If you were thinking of… | And then you find... | | This hypothesis becomes... | | | | | |
| B 28 | | | Narcolepsy | the patient had fallen several times for no reason | | -2 | -1 | 0 | +1 | +2 |  |
| B 29 | | | Epilepsy | had two febrile convulsions as a child | | -2 | -1 | 0 | +1 | +2 |  |
| B 30 | | | Postural orthostatic tachycardia syndrome | the patient does not suffer from vertigo | | -2 | -1 | 0 | +1 | +2 |  |
| -2 | Ruled out or almost ruled out | | |  |  |  |  |  |  |  |  |
| -1 | Less probable | | |  |  |  |  |  |  |  |  |
| 0 | Neither less or more probable | | |  |  |  |  |  |  |  |  |
| +1 | More probable | | |  |  |  |  |  |  |  |  |
| +2 | Certain or almost certain | | |  |  |  |  |  |  |  |  |
| **Case vignette 11:**  **A 38-year-old woman lost consciousness in the supermarket and fell onto the trolley. Lying on the floor, she briefly "twitched" and did not regain consciousness immediately afterwards. Now she has a laceration above the right eye.** | | | | | | | | | | | |
| Item number | | | If you were thinking of… | And then you find... | | This hypothesis becomes... | | | | | |
| B 31 | | | Convulsive syncope | the patient repetitively twitched on the left side of her face | | -2 | -1 | 0 | +1 | +2 |  |
| B 32 | | | Generalized epilepsy | the patient continues to complain of numbness of the left hand | | -2 | -1 | 0 | +1 | +2 |  |
| B 33 | | | Combined focal and generalized epilepsy | the patient had asymmetric extending movements of the extremity muscles | | -2 | -1 | 0 | +1 | +2 |  |
| -2 | Ruled out or almost ruled out | | |  |  |  |  |  |  |  |  |
| -1 | Less probable | | |  |  |  |  |  |  |  |  |
| 0 | Neither less or more probable | | |  |  |  |  |  |  |  |  |
| +1 | More probable | | |  |  |  |  |  |  |  |  |
| +2 | Certain or almost certain | | |  |  |  |  |  |  |  |  |

| **Case vignette 12:**  **A 20-year-old young man brings his 48-year-old father to the emergency room. He had found the father on the floor next to the kitchen table at home. The father is very tired and cannot give any information himself.** | | | | | | | | | | | |
| --- | --- | --- | --- | --- | --- | --- | --- | --- | --- | --- | --- |
| Item number | | | If you were thinking of… | And then you find... | | This hypothesis becomes... | | | | | |
| B 34 | | | Orthostatic hypotension | a Foetor alcoholicus is present | | -2 | -1 | 0 | +1 | +2 |  |
| B 35 | | | Reflex syncope | the GCS is at 9 points | | -2 | -1 | 0 | +1 | +2 |  |
| B 36 | | | Intoxication | a heart rate of 38/min exists | | -2 | -1 | 0 | +1 | +2 |  |
| -2 | Ruled out or almost ruled out | | |  |  |  |  |  |  |  |  |
| -1 | Less probable | | |  |  |  |  |  |  |  |  |
| 0 | Neither less or more probable | | |  |  |  |  |  |  |  |  |
| +1 | More probable | | |  |  |  |  |  |  |  |  |
| +2 | Certain or almost certain | | |  |  |  |  |  |  |  |  |

| **Case vignette 13:**  **A 32-year-old patient is admitted for evaluation with the suspicion of vasovagal syncope. Recently, she had increased problems with the circulation. In addition, a feeling of heat and nausea is described.** | | | | | | | | | | | |
| --- | --- | --- | --- | --- | --- | --- | --- | --- | --- | --- | --- |
| Item number | | | If you were considering to ask... | And then you find... | | This investigation becomes... | | | | | |
| B 37 | | | To perform the Schellong test | the patient is in the 18th week of pregnancy | | -2 | -1 | 0 | +1 | +2 |  |
| B 38 | | | To perform a 12-lead resting ECG | The patient has a history of dilated cardiomyopathy | | -2 | -1 | 0 | +1 | +2 |  |
| B 39 | | | To check the muscle reflexes | The patient has multiple sclerosis | | -2 | -1 | 0 | +1 | +2 |  |
| -2 | Completely or almost completely unnecessary | | |  |  |  |  |  |  |  |  |
| -1 | Less useful | | |  |  |  |  |  |  |  |  |
| 0 | Neither more nor less useful | | |  |  |  |  |  |  |  |  |
| +1 | More useful | | |  |  |  |  |  |  |  |  |
| +2 | Completely or almost completely necessary | | |  |  |  |  |  |  |  |  |

| **Case vignette 14:**  **An 18-year-old young woman was brought to the neurological outpatient clinic with a suspected epileptic seizure. Her friends report an acute onset of unconsciousness with tonic-clonic movements and enuresis. As you perform the clinical examination, you notice that the student is still somnolent.** | | | | | | | | | | | |
| --- | --- | --- | --- | --- | --- | --- | --- | --- | --- | --- | --- |
| Item number | | | If you were considering to ask... | And then you find... | | This investigation becomes... | | | | | |
| B 40 | | | To perform a blood gas analysis | the young woman was in a shisha bar with her friends | | -2 | -1 | 0 | +1 | +2 |  |
| B 41 | | | To order an EEG | the patient's parents are alcoholics | | -2 | -1 | 0 | +1 | +2 |  |
| B 42 | | | To order a CCT | the patient initially fell from a chair | | -2 | -1 | 0 | +1 | +2 |  |
| -2 | Completely or almost completely unnecessary | | |  |  |  |  |  |  |  |  |
| -1 | Less useful | | |  |  |  |  |  |  |  |  |
| 0 | Neither more nor less useful | | |  |  |  |  |  |  |  |  |
| +1 | More useful | | |  |  |  |  |  |  |  |  |
| +2 | Completely or almost completely necessary | | |  |  |  |  |  |  |  |  |

| **Case vignette 15:**  **An 85-year-old female retiree is admitted to the emergency department with dyspnea and lip cyanosis. The simplified Wells score was rated 2, so pulmonary artery embolism is suspected.** | | | | | | | | | | | |
| --- | --- | --- | --- | --- | --- | --- | --- | --- | --- | --- | --- |
| Item number | | | If you were considering to ask... | And then you find... | | This investigation becomes... | | | | | |
| B 43 | | | To determine the D-dimers | the patient is taking Marcumar | | -2 | -1 | 0 | +1 | +2 |  |
| B 44 | | | To undertake a pulmonary angiography | the patient is taking Thiamazole | | -2 | -1 | 0 | +1 | +2 |  |
| B 45 | | | To order an ECG | the patient has a pacemaker | | -2 | -1 | 0 | +1 | +2 |  |
| -2 | Completely or almost completely unnecessary | | |  |  |  |  |  |  |  |  |
| -1 | Less useful | | |  |  |  |  |  |  |  |  |
| 0 | Neither more nor less useful | | |  |  |  |  |  |  |  |  |
| +1 | More useful | | |  |  |  |  |  |  |  |  |
| +2 | Completely or almost completely necessary | | |  |  |  |  |  |  |  |  |

| **Case vignette 16:**  **You perform an examination of an unconscious patient. You consider an increase in intracranial pressure as the cause of the disturbance of consciousness.** | | | | | | | | | | | |
| --- | --- | --- | --- | --- | --- | --- | --- | --- | --- | --- | --- |
| Item number | | | If you were considering to ask... | And then you find... | | This investigation becomes... | | | | | |
| B 46 | | | To examine the brainstem reflexes | the patient makes specific defensive movements | | -2 | -1 | 0 | +1 | +2 |  |
| B 47 | | | To calculate the Glasgow Coma Scale | the eyes are opened to speech | | -2 | -1 | 0 | +1 | +2 |  |
| B 48 | | | To check for meningismus | there is an idiopathic Parkinson's syndrome | | -2 | -1 | 0 | +1 | +2 |  |
| -2 | Completely or almost completely unnecessary | | |  |  |  |  |  |  |  |  |
| -1 | Less useful | | |  |  |  |  |  |  |  |  |
| 0 | Neither more nor less useful | | |  |  |  |  |  |  |  |  |
| +1 | More useful | | |  |  |  |  |  |  |  |  |
| +2 | Completely or almost completely necessary | | |  |  |  |  |  |  |  |  |

| **Case vignette 17:**  **A 27-year-old female patient comes to the neurology department with externally diagnosed generalized tonic-clonic seizure. An EEG is ordered, which shows spike-wave patterns. This was the second seizure within the last four months.** | | | | | | | | | | | |
| --- | --- | --- | --- | --- | --- | --- | --- | --- | --- | --- | --- |
| Item number | | | If you were considering to prescribe... | And then you find... | | This prescription becomes... | | | | | |
| B 49 | | | To give Valproate | the patient wishes to have a child | | -2 | -1 | 0 | +1 | +2 |  |
| B 50 | | | The increase the preexisting Lamotrigine medication | the patient is taking the birth control pill | | -2 | -1 | 0 | +1 | +2 |  |
| B 51 | | | To give Levetiracetam | the patient has an impulse control disorder | | -2 | -1 | 0 | +1 | +2 |  |
| -2 | Completely or almost completely unnecessary | | |  |  |  |  |  |  |  |  |
| -1 | Less useful | | |  |  |  |  |  |  |  |  |
| 0 | Neither more nor less useful | | |  |  |  |  |  |  |  |  |
| +1 | More useful | | |  |  |  |  |  |  |  |  |
| +2 | Completely or almost completely necessary | | |  |  |  |  |  |  |  |  |

| **Case vignette 18:**  **A 44-year-old female patient is brought to the hospital by the ambulance. She had a focal seizure event lasting more than 10 minutes. It came along with a tremor of the left arm. After breaking through the seizure according to the therapy guideline, the patient is admitted to the epileptology department.** | | | | | | | | | | | |
| --- | --- | --- | --- | --- | --- | --- | --- | --- | --- | --- | --- |
| Item number | | | If you were considering to prescribe... | And then you find... | | This prescription becomes... | | | | | |
| B 52 | | | To undertake surgery | there is a focal finding in the EEG after a right cortical intracerebral hemorrhage | | -2 | -1 | 0 | +1 | +2 |  |
| B 53 | | | To start an anticonvulsant drug therapy | there is an inconspicuous provocation EEG in the patient file | | -2 | -1 | 0 | +1 | +2 |  |
| B 54 | | | To administer Cortisone | an right cerebral edema with an unclear mass on CT | | -2 | -1 | 0 | +1 | +2 |  |
| -2 | Completely or almost completely unnecessary | | |  |  |  |  |  |  |  |  |
| -1 | Less useful | | |  |  |  |  |  |  |  |  |
| 0 | Neither more nor less useful | | |  |  |  |  |  |  |  |  |
| +1 | More useful | | |  |  |  |  |  |  |  |  |
| +2 | Completely or almost completely necessary | | |  |  |  |  |  |  |  |  |

| **Case vignette 19:**  **A 57-year-old patient is on the neurology ward due his Wernicke's encephalopathy. He has not left his bed for several days. Now, tachycardia developed since the morning and the patient loses consciousness. They suspect a pulmonary artery embolism.** | | | | | | | | | | | |
| --- | --- | --- | --- | --- | --- | --- | --- | --- | --- | --- | --- |
| Item number | | | If you were considering to prescribe... | And then you find... | | This prescription becomes... | | | | | |
| B 55 | | | To administer Dabigatran | the liver values are strongly increased | | -2 | -1 | 0 | +1 | +2 |  |
| B 56 | | | To administer thrombolytic therapy | the patient requires resuscitation now | | -2 | -1 | 0 | +1 | +2 |  |
| B 57 | | | To administer Heparin | an existing thrombosis prophylaxis | | -2 | -1 | 0 | +1 | +2 |  |
| -2 | Completely or almost completely unnecessary | | |  |  |  |  |  |  |  |  |
| -1 | Less useful | | |  |  |  |  |  |  |  |  |
| 0 | Neither more nor less useful | | |  |  |  |  |  |  |  |  |
| +1 | More useful | | |  |  |  |  |  |  |  |  |
| +2 | Completely or almost completely necessary | | |  |  |  |  |  |  |  |  |

| **Case vignette 20:**  **A 24-year-old female patient is admitted to the emergency department with two self-limiting seizure events. She is treated by you the next day. Imaging and an EEG are unremarkable. You consider starting permanent anticonvulsive therapy.** | | | | | | | | | | | |
| --- | --- | --- | --- | --- | --- | --- | --- | --- | --- | --- | --- |
| Item number | | | If you were considering to prescribe... | And then you find... | | This prescription becomes... | | | | | |
| B 58 | | | To administer Levetiracetam | The patient has a post-traumatic stress disorder | | -2 | -1 | 0 | +1 | +2 |  |
| B 59 | | | To administer Tavor | The patient has an anxiety disorder | | -2 | -1 | 0 | +1 | +2 |  |
| B 60 | | | To administer Lamotrigine | the patient has a poor compliance | | -2 | -1 | 0 | +1 | +2 |  |
| -2 | Completely or almost completely unnecessary | | |  |  |  |  |  |  |  |  |
| -1 | Less useful | | |  |  |  |  |  |  |  |  |
| 0 | Neither more nor less useful | | |  |  |  |  |  |  |  |  |
| +1 | More useful | | |  |  |  |  |  |  |  |  |
| +2 | Completely or almost completely necessary | | |  |  |  |  |  |  |  |  |

Case 2

| **Case vignette 1:**  **A 57-year-old roofer has just been taken to the emergency room. His colleague from the construction site reported that the man suddenly complained about headaches and dizziness. Shortly thereafter, he had not spoken a word.** | | | | | | | | | | | |
| --- | --- | --- | --- | --- | --- | --- | --- | --- | --- | --- | --- |
| Item number | | | If you were thinking of… | And then you find... | | This hypothesis becomes... | | | | | |
| B 61 | | | Ischemic stroke | the patient is taking a statin | | -2 | -1 | 0 | +1 | +2 |  |
| B 62 | | | Intracerebral hemorrhage | the patient's blood pressure is 146/78 mmHg | | -2 | -1 | 0 | +1 | +2 |  |
| B 63 | | | Subarachnoid hemorrhage | the patient did not show up at work last week because of a severe headache | | -2 | -1 | 0 | +1 | +2 |  |
| -2 | Ruled out or almost ruled out | | |  |  |  |  |  |  |  |  |
| -1 | Less probable | | |  |  |  |  |  |  |  |  |
| 0 | Neither less or more probable | | |  |  |  |  |  |  |  |  |
| +1 | More probable | | |  |  |  |  |  |  |  |  |
| +2 | Certain or almost certain | | |  |  |  |  |  |  |  |  |

| **Case vignette 2:**  **After waking up, a 25-year-old female student noticed a paresis of the left arm. As a result, she immediately visited the outpatient clinic of the hospital.** | | | | | | | | | | | |
| --- | --- | --- | --- | --- | --- | --- | --- | --- | --- | --- | --- |
| Item number | | | If you were thinking of… | And then you find... | | This hypothesis becomes... | | | | | |
| B 64 | | | Stroke | the patient's mother has a Factor V Leiden mutation | | -2 | -1 | 0 | +1 | +2 |  |
| B 65 | | | Radial nerve paresis | the patient was at a student party the night before | | -2 | -1 | 0 | +1 | +2 |  |
| B 66 | | | Multiple sclerosis | the patient had an inflammation of the optic nerve two years ago | | -2 | -1 | 0 | +1 | +2 |  |
| -2 | Ruled out or almost ruled out | | |  |  |  |  |  |  |  |  |
| -1 | Less probable | | |  |  |  |  |  |  |  |  |
| 0 | Neither less or more probable | | |  |  |  |  |  |  |  |  |
| +1 | More probable | | |  |  |  |  |  |  |  |  |
| +2 | Certain or almost certain | | |  |  |  |  |  |  |  |  |

| **Case vignette 3:**  **A 38-year-old man notices a paresis of the right arm. In addition, his wife notices a drooping corner of the mouth on the right. They suspect an ischemic stroke.** | | | | | | | | | | | |
| --- | --- | --- | --- | --- | --- | --- | --- | --- | --- | --- | --- |
| Item number | | | If you were thinking of… | And then you find... | | This hypothesis becomes... | | | | | |
| B 67 | | | Territorial infarction | no sensory disturbances and no aphasia are present | | -2 | -1 | 0 | +1 | +2 |  |
| B 68 | | | Border zone infarction | the HBA1c at the last check-up was at 9.8% | | -2 | -1 | 0 | +1 | +2 |  |
| B 69 | | | Lacunar infarction | the patient has 50 pack years | | -2 | -1 | 0 | +1 | +2 |  |
| -2 | Ruled out or almost ruled out | | |  |  |  |  |  |  |  |  |
| -1 | Less probable | | |  |  |  |  |  |  |  |  |
| 0 | Neither less or more probable | | |  |  |  |  |  |  |  |  |
| +1 | More probable | | |  |  |  |  |  |  |  |  |
| +2 | Certain or almost certain | | |  |  |  |  |  |  |  |  |

| **Case vignette 4:**  **A 18-year-old female patient complains about an unilateral headache and dizziness. When the ambulance arrives, a paresis of the left arm is noticed.** | | | | | | | | | | | |
| --- | --- | --- | --- | --- | --- | --- | --- | --- | --- | --- | --- |
| Item number | | | If you were thinking of… | And then you find... | | This hypothesis becomes... | | | | | |
| B 70 | | | Sinus venous thrombosis | the patient is on birth control pill and smokes | | -2 | -1 | 0 | +1 | +2 |  |
| B 71 | | | Migraine with aura | the patient is not known to have migraines | | -2 | -1 | 0 | +1 | +2 |  |
| B 72 | | | Stroke | the paramedic has seen an arrhythmia in the ECG | | -2 | -1 | 0 | +1 | +2 |  |
| -2 | Ruled out or almost ruled out | | |  |  |  |  |  |  |  |  |
| -1 | Less probable | | |  |  |  |  |  |  |  |  |
| 0 | Neither less or more probable | | |  |  |  |  |  |  |  |  |
| +1 | More probable | | |  |  |  |  |  |  |  |  |
| +2 | Certain or almost certain | | |  |  |  |  |  |  |  |  |

| **Case vignette 5:**  **A 53-year-old man comes with recurrent, short-lasting visual disturbance in his right eye since three days. He reports that repeatedly "a wall builds up" for a short time and then he sees only "black". After a short period of time, this would be over again.** | | | | | | | | | | | |
| --- | --- | --- | --- | --- | --- | --- | --- | --- | --- | --- | --- |
| Item number | | | If you were thinking of… | And then you find... | | This hypothesis becomes... | | | | | |
| B 73 | | | Migraine | the patient reports flashes of light in front of the eyes | | -2 | -1 | 0 | +1 | +2 |  |
| B 74 | | | Internal carotid artery stenosis | Arteriosclerosis is not known in the history | | -2 | -1 | 0 | +1 | +2 |  |
| B 75 | | | Glaucoma | symptoms occur more frequently in the evening | | -2 | -1 | 0 | +1 | +2 |  |
| -2 | Ruled out or almost ruled out | | |  |  |  |  |  |  |  |  |
| -1 | Less probable | | |  |  |  |  |  |  |  |  |
| 0 | Neither less or more probable | | |  |  |  |  |  |  |  |  |
| +1 | More probable | | |  |  |  |  |  |  |  |  |
| +2 | Certain or almost certain | | |  |  |  |  |  |  |  |  |

| **Case vignette 6:**  **A 65-year-old pensioner comes to the emergency department. His wife reports that her husband was reading the newspaper at breakfast. Then all of a sudden his right arm became weak and he was talking gibberish.** | | | | | | | | | | | |
| --- | --- | --- | --- | --- | --- | --- | --- | --- | --- | --- | --- |
| Item number | | | If you were thinking of… | And then you find... | | This hypothesis becomes... | | | | | |
| B 76 | | | Cardioembolic stroke | the patient is taking apixaban (NOAK) | | -2 | -1 | 0 | +1 | +2 |  |
| B 77 | | | Macroangiopathic stroke | the patient has an internal carotid artery stenosis on the left side (50% according to NASCET) | | -2 | -1 | 0 | +1 | +2 |  |
| B 78 | | | Microangiopathic stroke | the patient is taking ramipril 5mg | | -2 | -1 | 0 | +1 | +2 |  |
| -2 | Ruled out or almost ruled out | | |  |  |  |  |  |  |  |  |
| -1 | Less probable | | |  |  |  |  |  |  |  |  |
| 0 | Neither less or more probable | | |  |  |  |  |  |  |  |  |
| +1 | More probable | | |  |  |  |  |  |  |  |  |
| +2 | Certain or almost certain | | |  |  |  |  |  |  |  |  |

| **Case vignette 7:**  **A 47-year-old automotive mechatronics technician sustained a humerus fracture requiring surgery at work. After the operation, a right leg paresis is noticed in the recovery room.** | | | | | | | | | | | |
| --- | --- | --- | --- | --- | --- | --- | --- | --- | --- | --- | --- |
| Item number | | | If you were thinking of… | And then you find... | | This hypothesis becomes... | | | | | |
| B 79 | | | Ischemic stroke | a persistent foramen ovale exists | | -2 | -1 | 0 | +1 | +2 |  |
| B 80 | | | Positional damage | the patient was placed in a sitting position intraoperatively | | -2 | -1 | 0 | +1 | +2 |  |
| B 81 | | | Intracerebral hemorrhage | blood pressure drops occurred during anesthesia | | -2 | -1 | 0 | +1 | +2 |  |
| -2 | Ruled out or almost ruled out | | |  |  |  |  |  |  |  |  |
| -1 | Less probable | | |  |  |  |  |  |  |  |  |
| 0 | Neither less or more probable | | |  |  |  |  |  |  |  |  |
| +1 | More probable | | |  |  |  |  |  |  |  |  |
| +2 | Certain or almost certain | | |  |  |  |  |  |  |  |  |

| **Case vignette 8:**  **A 22-year-old female patient woke up with numbness on the right side of her body. The patient is severely overweight and has hypertension requiring treatment.** | | | | | | | | | | | |
| --- | --- | --- | --- | --- | --- | --- | --- | --- | --- | --- | --- |
| Item number | | | If you were thinking of… | And then you find... | | This hypothesis becomes... | | | | | |
| B 82 | | | Wake-Up Stroke | the FAST test is negative | | -2 | -1 | 0 | +1 | +2 |  |
| B 83 | | | Migraine | the patient has right-sided headaches now | | -2 | -1 | 0 | +1 | +2 |  |
| B 84 | | | Multiple sclerosis | an MRI of the skull was inconspicuous two weeks ago | | -2 | -1 | 0 | +1 | +2 |  |
| -2 | Ruled out or almost ruled out | | |  |  |  |  |  |  |  |  |
| -1 | Less probable | | |  |  |  |  |  |  |  |  |
| 0 | Neither less or more probable | | |  |  |  |  |  |  |  |  |
| +1 | More probable | | |  |  |  |  |  |  |  |  |
| +2 | Certain or almost certain | | |  |  |  |  |  |  |  |  |

| **Case vignette 9:**  **A 72-year-old patient reports about recurrent weakness of the right arm for two weeks. Strength testing shows a normal finding.** | | | | | | | | | | | |
| --- | --- | --- | --- | --- | --- | --- | --- | --- | --- | --- | --- |
| Item number | | | If you were thinking of… | And then you find... | | This hypothesis becomes... | | | | | |
| B 85 | | | Focal epileptic seizure | sensory disturbances have never occurred before | | -2 | -1 | 0 | +1 | +2 |  |
| B 86 | | | Recurrent transient ischemic attacks | the MRI of the skull is inconspicuous | | -2 | -1 | 0 | +1 | +2 |  |
| B 87 | | | Internal carotid artery stenosis on the left side | the patient's wife has noticed a drooping corner of the mouth on the right side several times | | -2 | -1 | 0 | +1 | +2 |  |
| -2 | Ruled out or almost ruled out | | |  |  |  |  |  |  |  |  |
| -1 | Less probable | | |  |  |  |  |  |  |  |  |
| 0 | Neither less or more probable | | |  |  |  |  |  |  |  |  |
| +1 | More probable | | |  |  |  |  |  |  |  |  |
| +2 | Certain or almost certain | | |  |  |  |  |  |  |  |  |

| **Case vignette 10:**  **A 45-year-old female patient complains about acute dizziness with nausea and vomiting. She had already been feeling unwell for the last two days.** | | | | | | | | | | | |
| --- | --- | --- | --- | --- | --- | --- | --- | --- | --- | --- | --- |
| Item number | | | If you were thinking of… | And then you find... | | This hypothesis becomes... | | | | | |
| B 88 | | | Neuritis vestibularis | has a rotatory spontaneous nystagmus | | -2 | -1 | 0 | +1 | +2 |  |
| B 89 | | | Progressive stroke | the patient has a colon cancer | | -2 | -1 | 0 | +1 | +2 |  |
| B 90 | | | Basilar artery thrombosis | the patient has bilateral ataxic pointing tests | | -2 | -1 | 0 | +1 | +2 |  |
| -2 | Ruled out or almost ruled out | | |  |  |  |  |  |  |  |  |
| -1 | Less probable | | |  |  |  |  |  |  |  |  |
| 0 | Neither less or more probable | | |  |  |  |  |  |  |  |  |
| +1 | More probable | | |  |  |  |  |  |  |  |  |
| +2 | Certain or almost certain | | |  |  |  |  |  |  |  |  |

| **Case vignette 11:**  **A 44-year-old father has come to the outpatient department of a hospital with his daughter. When the girl is sent for to the radiology department, the father's newspaper falls out of his hand and he notices diplopia.** | | | | | | | | | | | |
| --- | --- | --- | --- | --- | --- | --- | --- | --- | --- | --- | --- |
| Item number | | | If you were considering to ask... | And then you find... | | This investigation becomes... | | | | | |
| B 91 | | | To investigate the finger-nose test | the patient has a known myasthenia gravis | | -2 | -1 | 0 | +1 | +2 |  |
| B 92 | | | To be examined according to the BEFAST scheme | the patient also reports dizziness | | -2 | -1 | 0 | +1 | +2 |  |
| B 93 | | | To calculate the NIHSS | the same symptomatology has already occurred in the morning for a short time | | -2 | -1 | 0 | +1 | +2 |  |
| -2 | Completely or almost completely unnecessary | | |  |  |  |  |  |  |  |  |
| -1 | Less useful | | |  |  |  |  |  |  |  |  |
| 0 | Neither more nor less useful | | |  |  |  |  |  |  |  |  |
| +1 | More useful | | |  |  |  |  |  |  |  |  |
| +2 | Completely or almost completely necessary | | |  |  |  |  |  |  |  |  |

| **Case vignette 12:**  **A 55-year-old patient gets to the emergency department with right brachiofacial hemiparesis and aphasia.** | | | | | | | | | | | |
| --- | --- | --- | --- | --- | --- | --- | --- | --- | --- | --- | --- |
| Item number | | | If you were considering to ask... | And then you find... | | This investigation becomes... | | | | | |
| B 94 | | | To order a cMRI | the onset of symptoms is not clear yet | | -2 | -1 | 0 | +1 | +2 |  |
| B 95 | | | To order a cCT with a contrast medium | the patient has a creatinine level of 1.4 | | -2 | -1 | 0 | +1 | +2 |  |
| B 96 | | | To do a doppler sonography | a 60% stenosis of the left internal carotid artery exists | | -2 | -1 | 0 | +1 | +2 |  |
| -2 | Completely or almost completely unnecessary | | |  |  |  |  |  |  |  |  |
| -1 | Less useful | | |  |  |  |  |  |  |  |  |
| 0 | Neither more nor less useful | | |  |  |  |  |  |  |  |  |
| +1 | More useful | | |  |  |  |  |  |  |  |  |
| +2 | Completely or almost completely necessary | | |  |  |  |  |  |  |  |  |

| **Case vignette 13:**  **A 83-year-old female patient comes to the emergency department with a suspected right-sided infarction of the middle cerebral artery.** | | | | | | | | | | | |
| --- | --- | --- | --- | --- | --- | --- | --- | --- | --- | --- | --- |
| Item number | | | If you were considering to ask... | And then you find... | | This investigation becomes... | | | | | |
| B 97 | | | To determine the coagulation parameters | the symptoms started only one hour ago | | -2 | -1 | 0 | +1 | +2 |  |
| B 98 | | | To determine the cholesterol levels | the patient is taking a statin | | -2 | -1 | 0 | +1 | +2 |  |
| B 99 | | | To determine the troponin levels | the patient does not have angina pectoris | | -2 | -1 | 0 | +1 | +2 |  |
| -2 | Completely or almost completely unnecessary | | |  |  |  |  |  |  |  |  |
| -1 | Less useful | | |  |  |  |  |  |  |  |  |
| 0 | Neither more nor less useful | | |  |  |  |  |  |  |  |  |
| +1 | More useful | | |  |  |  |  |  |  |  |  |
| +2 | Completely or almost completely necessary | | |  |  |  |  |  |  |  |  |

| **Case vignette 14:**  **You are on duty in the stroke unit. A patient was given thrombolysis intravenously in the morning. The reason was an ischemic stroke with right hemiparesis and word finding problems. For about 15 minutes, the patient has been globally aphasic and has a head gaze to the left.** | | | | | | | | | | | |
| --- | --- | --- | --- | --- | --- | --- | --- | --- | --- | --- | --- |
| Item number | | | If you were considering to ask... | And then you find... | | This investigation becomes... | | | | | |
| B 100 | | | To order a native CT | the patient has had an peak blood pressure of 200/110 mmHg | | -2 | -1 | 0 | +1 | +2 |  |
| B 101 | | | To order a CT perfusion imaging | the patient has untreated atrial fibrillation | | -2 | -1 | 0 | +1 | +2 |  |
| B 102 | | | To order an EEG | the patient was temporarily unconscious at the onset of his current symptoms | | -2 | -1 | 0 | +1 | +2 |  |
| -2 | Completely or almost completely unnecessary | | |  |  |  |  |  |  |  |  |
| -1 | Less useful | | |  |  |  |  |  |  |  |  |
| 0 | Neither more nor less useful | | |  |  |  |  |  |  |  |  |
| +1 | More useful | | |  |  |  |  |  |  |  |  |
| +2 | Completely or almost completely necessary | | |  |  |  |  |  |  |  |  |

| **Case vignette 15:**  **A 68-year-old patient in the stroke unit is clinically suspected of having an**  **internal carotid artery stenosis.** | | | | | | | | | | | |
| --- | --- | --- | --- | --- | --- | --- | --- | --- | --- | --- | --- |
| Item number | | | If you were considering to ask... | And then you find... | | This investigation becomes... | | | | | |
| B 103 | | | To do a duplex sonography | the patient received an internal carotid artery stent two years ago | | -2 | -1 | 0 | +1 | +2 |  |
| B 104 | | | To order an MRI angiography | the patient suffers from claustrophobia | | -2 | -1 | 0 | +1 | +2 |  |
| B 105 | | | To order a CT perfusion imaging | the patient has poor venous conditions and a 22 gauge (blue) venous access | | -2 | -1 | 0 | +1 | +2 |  |
| -2 | Completely or almost completely unnecessary | | |  |  |  |  |  |  |  |  |
| -1 | Less useful | | |  |  |  |  |  |  |  |  |
| 0 | Neither more nor less useful | | |  |  |  |  |  |  |  |  |
| +1 | More useful | | |  |  |  |  |  |  |  |  |
| +2 | Completely or almost completely necessary | | |  |  |  |  |  |  |  |  |

| **Case vignette 16:**  **A 32-year-old office administrator has been suffering from migraine attacks for years. Today, she is admitted to the emergency room with numbness, weakness of the right arm for an hour and word-finding problems.** | | | | | | | | | | |
| --- | --- | --- | --- | --- | --- | --- | --- | --- | --- | --- |
| Item number | | | If you were considering to prescribe... | And then you find... | | This prescription becomes... | | | | |
| B 106 | | | To classify the patient in the triage system as blue | the patient also has high blood pressure | | -2 | -1 | 0 | +1 | +2 |
| B 107 | | | To give a triptan | migraine aura has never occurred before | | -2 | -1 | 0 | +1 | +2 |
| B 108 | | | To administer thrombolytic therapy | the patient had similar aura phenomena before | | -2 | -1 | 0 | +1 | +2 |
| -2 | Completely or almost completely unnecessary | | |  |  |  |  |  |  |  |
| -1 | Less useful | | |  |  |  |  |  |  |  |
| 0 | Neither more nor less useful | | |  |  |  |  |  |  |  |
| +1 | More useful | | |  |  |  |  |  |  |  |
| +2 | Completely or almost completely necessary | | |  |  |  |  |  |  |  |

| **Case vignette 17:**  **A 72-year-old man noticed right mouth weakness 35 minutes ago. It is getting harder for him to speak now. The native CT shows no early signs of infarction. Contrast-enhanced CT imaging shows a vessel rupture in the area of the left cerebri medial artery (M2 branch).** | | | | | | | | | | |
| --- | --- | --- | --- | --- | --- | --- | --- | --- | --- | --- |
| Item number | | | If you were considering to prescribe... | And then you find... | | This prescription becomes... | | | | |
| B 109 | | | To undertake a carotid endarterectomy | there is a left internal carotid artery stenosis of 50% according to NASCET as well | | -2 | -1 | 0 | +1 | +2 |
| B 110 | | | To undertake a thrombolytic therapy | the patient had a prostate surgery last week | | -2 | -1 | 0 | +1 | +2 |
| B 111 | | | To undertake an interventional thrombectomy | the patient takes Marcumar irregularly | | -2 | -1 | 0 | +1 | +2 |
| -2 | Completely or almost completely unnecessary | | |  |  |  |  |  |  |  |
| -1 | Less useful | | |  |  |  |  |  |  |  |
| 0 | Neither more nor less useful | | |  |  |  |  |  |  |  |
| +1 | More useful | | |  |  |  |  |  |  |  |
| +2 | Completely or almost completely necessary | | |  |  |  |  |  |  |  |

| **Case vignette 18:**  **A 63-year-old man has been suffering from a dull-pressing headache, nausea and vomiting for three hours. Neurological examination shows a drop in the left arm grip. The blood pressure is 187/98 mmHg.** | | | | | | | | | | | |
| --- | --- | --- | --- | --- | --- | --- | --- | --- | --- | --- | --- |
| Item number | | | If you were considering to prescribe... | And then you find... | | This prescription becomes... | | | | | |
| B 112 | | | To undertake a thrombolytic therapy | native CT shows hyperdensity in the area of the right basal ganglia | | -2 | -1 | 0 | +1 | +2 |  |
| B 113 | | | To undertake an interventional thrombectomy | a hyperdense media sign is visualized on native CT | | -2 | -1 | 0 | +1 | +2 |  |
| B 114 | | | To reduce blood pressure to a target systolic of 120 mmHg | there is a large mismatch in the CT perfusion imaging | | -2 | -1 | 0 | +1 | +2 |  |
| -2 | Completely or almost completely unnecessary | | |  |  |  |  |  |  |  |  |
| -1 | Less useful | | |  |  |  |  |  |  |  |  |
| 0 | Neither more nor less useful | | |  |  |  |  |  |  |  |  |
| +1 | More useful | | |  |  |  |  |  |  |  |  |
| +2 | Completely or almost completely necessary | | |  |  |  |  |  |  |  |  |

| **Case vignette 19:**  **A 79-year-old female patient comes with an ambulance service. She has had right hemiparesis for 40 minutes and is globally aphasic. CT angiography with contrast medium shows occlusion of the M1 branch of the left middle cerebral artery.** | | | | | | | | | | |
| --- | --- | --- | --- | --- | --- | --- | --- | --- | --- | --- |
| Item number | | | If you were considering to prescribe... | And then you find... | | This prescription becomes... | | | | |
| B 115 | | | To undertake thrombolytic therapy | the patient received a knee replacement 16 days ago | | -2 | -1 | 0 | +1 | +2 |
| B 116 | | | To undertake an interventional thrombectomy | symptoms declined in the shock room | | -2 | -1 | 0 | +1 | +2 |
| B 117 | | | To undertake thrombolytic therapy | the patient is taking dabigatran (NOAK) for atrial fibrillation | | -2 | -1 | 0 | +1 | +2 |
| -2 | Completely or almost completely unnecessary | | |  |  |  |  |  |  |  |
| -1 | Less useful | | |  |  |  |  |  |  |  |
| 0 | Neither more nor less useful | | |  |  |  |  |  |  |  |
| +1 | More useful | | |  |  |  |  |  |  |  |
| +2 | Completely or almost completely necessary | | |  |  |  |  |  |  |  |

| **Case vignette 20:**  **A 41-year-old patient is presented by the ambulance service with decreased vigilance. Clinical examination shows a decreased tone in the right arm.** | | | | | | | | | | | |
| --- | --- | --- | --- | --- | --- | --- | --- | --- | --- | --- | --- |
| Item number | | | If you were considering to prescribe... | And then you find... | | This prescription becomes... | | | | | |
| B 118 | | | To start a secondary prophylaxis with ASA and atorvastatin | the serum glucose is at 43 mg/dl | | -2 | -1 | 0 | +1 | +2 |  |
| B 119 | | | To undertake a thrombolytic therapy | the blood pressure is 90/45 mmHg and the heart rate is 136/min | | -2 | -1 | 0 | +1 | +2 |  |
| B 120 | | | To start volume therapy | the patient's body temperature is 38.7°C | | -2 | -1 | 0 | +1 | +2 |  |
| -2 | Completely or almost completely unnecessary | | |  |  |  |  |  |  |  |  |
| -1 | Less useful | | |  |  |  |  |  |  |  |  |
| 0 | Neither more nor less useful | | |  |  |  |  |  |  |  |  |
| +1 | More useful | | |  |  |  |  |  |  |  |  |
| +2 | Completely or almost completely necessary | | |  |  |  |  |  |  |  |  |

**Examination Part C: Questionnaire**

Personal Code:

| Nr. | Question |
| --- | --- |
| C 1 | Please indicate your age: _______ years |
| C 2 | Do you have any prior medical training (e.g., nursing, emergency medical services, physical therapy, or similar)?   - Yes - No   If „Yes“: Please indicate which:: ________________________________ |
| C 3 | In which specialty are you completing your PJ elective?  __________________________ |
| C 4 | In which specialty would you like to do your advanced specialty training?  __________________________ |
| C 5 | Have you had any experience with digital, interactive teaching formats (e.g. "serious games") during your medical studies?   - Yes - No - Don’t know   If "yes": Which format have you already worked with?  ___________________________ |
| C 6 | Which medium do you primarily use to study for medical school?  (Please indicate percentage - 100% should be reached at the end)  Learning platforms on the Internet ____________% (Please specify which: ______________)  Books ____________%  Podcasts ____________%  Radio/TV ____________%  Others ____________% (Please specify which: ______________)  Others ____________% (Please specify which: ______________) |
| C 7 | Are you using learning groups in the development of practical subject areas?   - Yes - No - Don’t know |
| C 8 | How do you rate the Script Concordance Test compared to the common multiple choice tests?  ________________________________________________________________________________  ________________________________________________________________________________ |
| C 9 | **Please answer the following questions using the response options "Agree fully," "Agree," "Neutral," "Agree less," Disagree."**  **I learned in medical school how to make clinical decisions in emergency situations.**   - Fully agree - Agree - Neutral - Agree less - Disagree   **I had the opportunity to practice decision making in clinical emergency situations in medical school.**   - Fully agree - Agree - Neutral - Agree less - Disagree   **I feel confident in making clinical decisions in emergency situations.**   - Fully agree - Agree - Neutral - Agree less - Disagree   **The course helped me structuring my thoughts in a clinical emergency situation.**   - Fully agree - Agree - Neutral - Agree less - Disagree   **Throughout the course, I feel better prepared for clinical emergency situations.**  **I enjoyed the course.**   - Fully agree - Agree - Neutral - Agree less - Disagree   **I found the course and the case scenarios were difficult.**   - Fully agree - Agree - Neutral - Agree less - Disagree   **found the course and the case scenarios were exhausting.**   - Fully agree - Agree - Neutral - Agree less - Disagree   **The course and the questions put me under stress.**   - Stimme voll zu - Stimme zu - Neutral - Stimme weniger zu - Stimme nicht zu   **As a result of the course, I have more fear of clinical emergency situations.**   - Fully agree - Agree - Neutral - Agree less - Disagree   **As a result of the course, I have less fear of clinical emergency situations.**   - Fully agree - Agree - Neutral - Agree less - Disagree   **The course helped me to become aware of processes in the clinic.**   - Fully agree - Agree - Neutral - Agree less - Disagree   **The course is a good preparation for the first clinical services.**   - Fully agree - Agree - Neutral - Agree less - Disagree   **I was bored by the course.**   - Fully agree - Agree - Neutral - Agree less - Disagree   **The course has challenged me.**   - Fully agree - Agree - Neutral - Agree less - Disagree   **The course sparked my interest in neurology.**   - Fully agree - Agree - Neutral - Agree less - Disagree |
| C 10 | Free text (please briefly describe how you felt about the course):  How did you mentally approach answering the questions (e.g., "decided on gut instinct" or "drew on wealth of experience" )?  ______________________________________________________  ______________________________________________________  ______________________________________________________  ______________________________________________________  ______________________________________________________  How have you made clinical decisions so far? Has anything changed after taking the course?  ______________________________________________________  ______________________________________________________  ______________________________________________________  ______________________________________________________  ______________________________________________________  How did you like the course? Do you have any other comments or requests?  ______________________________________________________  ______________________________________________________  ______________________________________________________  ______________________________________________________  ______________________________________________________ |

**Thank you for participating in the study!**
